# A Core Omnigenic Non-coding Trait Governing Dex-Induced Osteoporotic Effects Identified Without DEXA

**DOI:** 10.3389/fphar.2021.750959

**Published:** 2021-11-24

**Authors:** Li Lu, Yanzhen Cai, Xiaoling Luo, Zhangting Wang, Sin Hang Fung, Huanhuan Jia, Chi Lam Yu, Wai Yee Chan, Kai Kei Miu, Wende Xiao

**Affiliations:** ^1^ Guangdong Key Laboratory of Pharmaceutical Bioactive Substances, School of Life Science and Biopharmacy, Guangdong Pharmaceutical University, Guangzhou, China; ^2^ School of Biomedical Sciences, Faculty of Medicine, The Chinese University of Hong Kong, Hong Kong, SAR China; ^3^ Guangdong Key Laboratory of Laboratory Animals, Guangdong Laboratory Animals Monitoring Institute, Guangzhou, China; ^4^ Department of Orthopedics, Guangzhou First People’s Hospital, School of Medicine, South China University of Technology, Guangzhou, China

**Keywords:** glucocorticoid, glucocorticoid-induced osteoporosis, bone loss, bone morphogenetic protein signaling pathway, osteoblasts differentiation, *cis*-expression quantitative trait loci

## Abstract

Iatrogenic glucocorticoid (GC)-induced osteoporosis (GIO) is an idiosyncratic form of secondary osteoporosis. Genetic predisposition among individuals may give rise to variant degree of phenotypic changes but there has yet been a documented unified pathway to explain the idiosyncrasy. In this study, we argue that the susceptibility to epigenetic changes governing molecular cross talks along the BMP and PI3K/Akt pathway may underline how genetic background dictate GC-induced bone loss. Concordantly, osteoblasts from BALB/c or C57BL/6 neonatal mice were treated with dexamethasone for transcriptome profiling. Furthermore, we also confirmed that GC-pre-conditioned mesenchymal stem cells (MSCs) would give rise to defective osteogenesis by instigating epigenetic changes which affected the accessibility of enhancer marks. In line with these epigenetic changes, we propose that GC modulates a key regulatory network involving the scavenger receptor Cd36 in osteoblasts pre-conditioning pharmacological idiosyncrasy in GIO.

## Introduction

Osteoporosis is a prevalent multifactorial metabolic bone disease characterized by diminished bone mineral density (BMD), staging a heightened risk of bone fracture in more than 200 million individuals worldwide (Sözen et al., 2017). Despite natural aging process would contribute to bone loss as in osteoporosis, disease medications—particularly steroid drugs—also contributed notably to the increased risk of such bone failure (Kenanidis et al., 2015). Leveraging arguments about predisposition from either environmental or genetic factors, it remains tempting to pinpoint genetics as fracture-related traits in osteoporosis (Zheng et al., 2011).

Glucocorticoids (GC) are celebrated for their prescriptive use in anti-inflammatory regimes suppressive in our body defense; therefore, they are useful in managing autoimmune diseases and viral infections (Becker, 2013; Robinson and Morand, 2021). One of its agonists, dexamethasone (Dex) was highlighted as the single life-saver medication for patients suffering cytokine syndrome in the current SARS-CoV-2 pandemic (Horby et al., 2021). Retrospectively, the incidence rates of both avascular necrosis of femoral head and of osteoporosis were found higher in convalescent SARS patients than in general population (Xie et al., 2005). Likewise, there is seemingly imminent advantage for GC administration in the treatment of rheumatoid arthritis, yet the symptomatic relief is disillusioned along with notorious appearance of bone deterioration which prevented their clinical use (Huber and Ward, 2016).

Furthermore, iatrogenic hypercortisolism is also named as a common side effect associated with GC treatment in managing lupus and psoriasis (Sachdeva et al., 2020; Mejía-Vilet and Ayoub, 2021). These treatment regimens would frequently demonstrate an idiosyncratic damage to bony structure, manifested in vertebral fractures classified as GIO (Briot and Roux, 2015). Clinical manifestations are apparently shared among GIO to other named causes for osteoporosis except for an early rapid bone loss occurred even when patients remained of a BMD levels slightly higher than those patients diagnosed with post-menopausal osteoporosis (Canalis et al., 2007). Furthermore, GC side-effects are idiosyncratic to denote only a meagre positive trend for femoral head necrosis along higher dosage of GC administered (Kameda et al., 2009; Sekiya et al., 2010; Chen et al., 2021). GCs exert their transcriptional effects by serving as the ligands for cytoplasmic glucocorticoid type 2 receptors (GCR) predominantly enriched in osteoblastic lineages (Dovio et al., 2004). With reference to measurable trabeculae loss in GIO, it is almost certain that GC would either restraint mineralization activity for osteoblasts themselves or would affect the efficacy of osteoblast differentiation from their precursor stromal cells.

The central goal of employing genetics model to study molecular etiology is to understand the links between genetic variation and disease outcomes (Witte, 2010). It was envisioned that GC drug efficacy and associated idiosyncratic effects could be examined either epidemiologically or in experimental model settings, wherein inbred mouse strains would provide the valuable tool to study possible genetic regulation of GC-induced bone loss. Unfortunately, little attention was put on safeguarding against phenotypes that might have arisen from subtle inbred line differences among common mouse strains (Yoshiki and Moriwaki, 2006).

In this study, we explored the genetic background in modulating GC-induced bone mass change by skeletal and cartilage response to Dex between BALB/c and C57BL/6 mice. Also, parallel comparison was conducted in gene expression for *ex vivo* osteoblast cultures isolated from either the Collaborative Cross (CC) mouse lines or conventional mouse lines, confirming that inherent genetic differences is sufficient to mask off the phenotypic heterogeneity in response to GC regardless of whether Dex was pre-conditioned. Furthermore, to understand individual’s susceptibility to GC-induce GIO risk resulted from genetic background, we compared the dynamic histone marks in MSC-derived osteoblast differentiation model with or without Dex pre-condition. Indeed, we revealed differential histone marker and protein expression changes in a gene of osteogenic core-like property CD36, which belonged to a co-expression network particularly vulnerable to trabeculae loss, interacting with genes of GWAS signatures predisposing fracture risks.

## Materials and Methods

### Animal and Experimental Design

Thirty-one 8-week-old C57BL/6Jnifdc (C57BL/6) mice and thirty 8-week-old BALB/cCrSlcNifdc (BALB/c) mice (Experimental Animal Center of Guangzhou University of Chinese Medicine, BALB/c: SCXK 2008-0002, C57BL/6: SCXK 2008-0002) were given 14 days of adaptation period with similar feeding and housing conditions prior to treatment to prevent impact from environmental variance. Mice were randomly divided into 4 groups, including the control group (Ctrl, non-treatment), Low-dose (Dex, 0.2 mg/kg four times per week by i. p.), Mid-dose (Dex, 0.5 mg/kg four times per week by i. p.) and High-dose (Dex, 1.0 mg/kg four times per week by i. p.). The administration period was 60 days in total. Each group of mice was housed individually in a room with a 12 h:12 h light/dark cycle with free access to complete pellet diets and water. All experimental procedures and animal care were approved by Animal Use and Care Committee of Guangdong Pharmaceutical University.

### Cell Culture and Differentiation

Mesenchymal Stem Cells (MSCs) were cultured in Dulbecco’s Modified Eagle’s medium (DMEM) containing 10% fetal bovine serum (FBS), 100 IU/ml penicillin, and 100 μg/ml streptomycin at 37°C in 5% CO2. Cells were passed twice a week. Differentiation of MSCs towards osteoblasts were performed according to previous protocols (Hanna et al., 2018).

### Drug Treatment and ChIP-Seq Analysis in Human Bone Marrow Mesenchymal Stem Cells

Human bone marrow MSCs of passage 8-10 were pre-conditioned with or without 1 μM μM concentration of Dex for 72 h followed by a subsequent 21-days osteogenic differentiation protocol by StemPro Osteogenesis Differentiation Kit according to manufacturer’s manual. For examining the enhancer marker changes instigated along osteogenesis, Dex- or vehicle-treated MSCs were subjected to the same 72 h pre-conditioning followed by a brief 24 h treatment in the osteogenic medium. These pro-osteogenic MSCs were then collected and subjected to Cleavage Under Targets and Tagmentation (CUT and Tag) chromatin immunoprecipitation profiling with mono-methylated H3K4 (H3K4me1) enhancer mark-specific antibody (Abcam-ab176877) along the standard protocol in accordance to the manufacturer’s manual enlisted in the hyperactive *In-Situ* ChIP library prep kit (Vazyme-TD902). Other antibodies used in the current research work are anti-GAPDH (#5174) and anti-NR3C1 (#3660) (Cell signaling technologies), anti-TAGLN3 (abcam-ab204902), anti-CD36 (abcam-ab133625) and anti-stabilin-1 (Santa Cruz sc-293254).

### Autopsy and Sample Preparation

All experimental subjects were labelled with calcein under double subcutaneous injection regime of 10 mg/kg per day for two consecutive days, 13, 14, 2 and 3 days before animal sacrifice. The proximal tibia metaphysis (PTM) and middle part of tibia shaft (TX) were taken for bone histomorphometry, anatomized to remove soft tissue, and then embedded in methyl methacrylate thereafter sectioned by hard tissue Microtome (Leica 2155, Germany). The slices were stained with Masson-Goldner Trichrome bone strain method described as previously reported (Foot, 1933).

### Bone Histomorphometric Analysis

Measurements were performed with a digital system consisting of fluorescent microscope with bright-field channels. The system was coupled to a computer with a morphometry program “Bioquant OSTEO 2009” (Bioquant Corporation, United States). Static parameters of trabecular bone, including the trabecular bone area percentage (%Tb.Ar) and trabecular thickness (Tb.Th), number (Tb.N), separation (Tb.Sp). For the cortical bone, parameters including total tissue area, percentage cortical area (%Ct.Ar), percentage marrow area (%Ma.Ar), cortical bone width and cortical bone endosteal formation rate (E-BFR/BS, a dynamic parameters). Label escape correction was used for the calculation of BFR (Jia et al., 2020).

### Osteoblast Culture and RNA Sequencing Analysis

Osteoblasts isolated from calvarias of 1-day-old C57BL/6 and BALB/c neonatal mice (Experimental Animal Center, Sun Yat-Sen University: 44008500012901) were cultured in MEM with 10% FBS and 100 U/mL penicillin, respectively. Cells were randomly divided into 2 groups, including Control group (MEM with 10% FBS and 100 U/mL penicillin) and Dex-treated group (Dex, 5*10^−7^M, MEM with 10% FBS and 100 U/mL penicillin). Passage 3 of osteoblasts were treated with Dex for 72 h.

Total cellular RNA was extracted and performed RNA sequencing (RNAseq). RNA-seq was performed using the BGISEQ-500 platform (BGI). The quality of sequencing reads was checked using the FastQC program (version 0.11.5). After adaptor trimming and removal of poor-quality reads (Q < 15) using SOAPnuke (SOAPnuke v1.5.2, BGI, China), Bowtie (bowtie2-v2.2.5) were used to map RNA-seq reads to mouse reference genome version mm10. After assembling reads to the genome using RSEM (RSEM, v1.2.12), gene expression was normalized against total read count (Fragment Per Kilobase of transcript per Million mapped reads, FPKM).

Differentially expressed genes (DEGs) in Dex-treated cells were determined based on Pearson’s correlation calculated using *cor* function of RSEM. Selection criteria for DEGs in this study were adjusted *p*-value (reported as false discovery rate in RSEM) ≤ 0.001 and fold change ≥2.0. For biological interpretation of DEGs, KEGG (the Kyoto Encyclopedia of Genes and Genomes) pathway analysis and Gene Ontology (GO) enrichment analysis of DEGs were performed using DAVID (the Database for Annotation, Visualization and Integrated Discovery, https://david.ncifcrf.gov/). For the meta-analysis of RNA-seq data for PCA analysis adapted for CC murine calvaria osteoblast culture from *Sabik et al.* (GSE134081) and osteogenic stromal progenitor culture from *Khayal et al.* (GSE54461), they were publicly-available from Gene Expression Omnibus database at the corresponding accession numbers.

### Statistical Analysis

Statistical analyses were performed using the Statistical Package for Social Sciences (SPSS 13.0; SPSS, Inc., Chicago, IL). Differences among groups were detected using one-way ANOVA. Values were expressed as mean ± SD, and the difference between groups was defined as significant at *p* < 0.05.

## Results

### Cross-Examination of Dex-Induced Cancellous Bone Loss in BALB/C and C57BL/6 Mice

We firstly demonstrated that various strains of inbred mice had differential sensitivity towards bone loss recapitulating features associated with GIO. Here, adult mouse from either C57BL/6 or BALB/c mice were administered with various dosages of Dex for 60 days, followed by subjecting their tibia to bone histomorphometric analysis.

**FIGURE 1:**
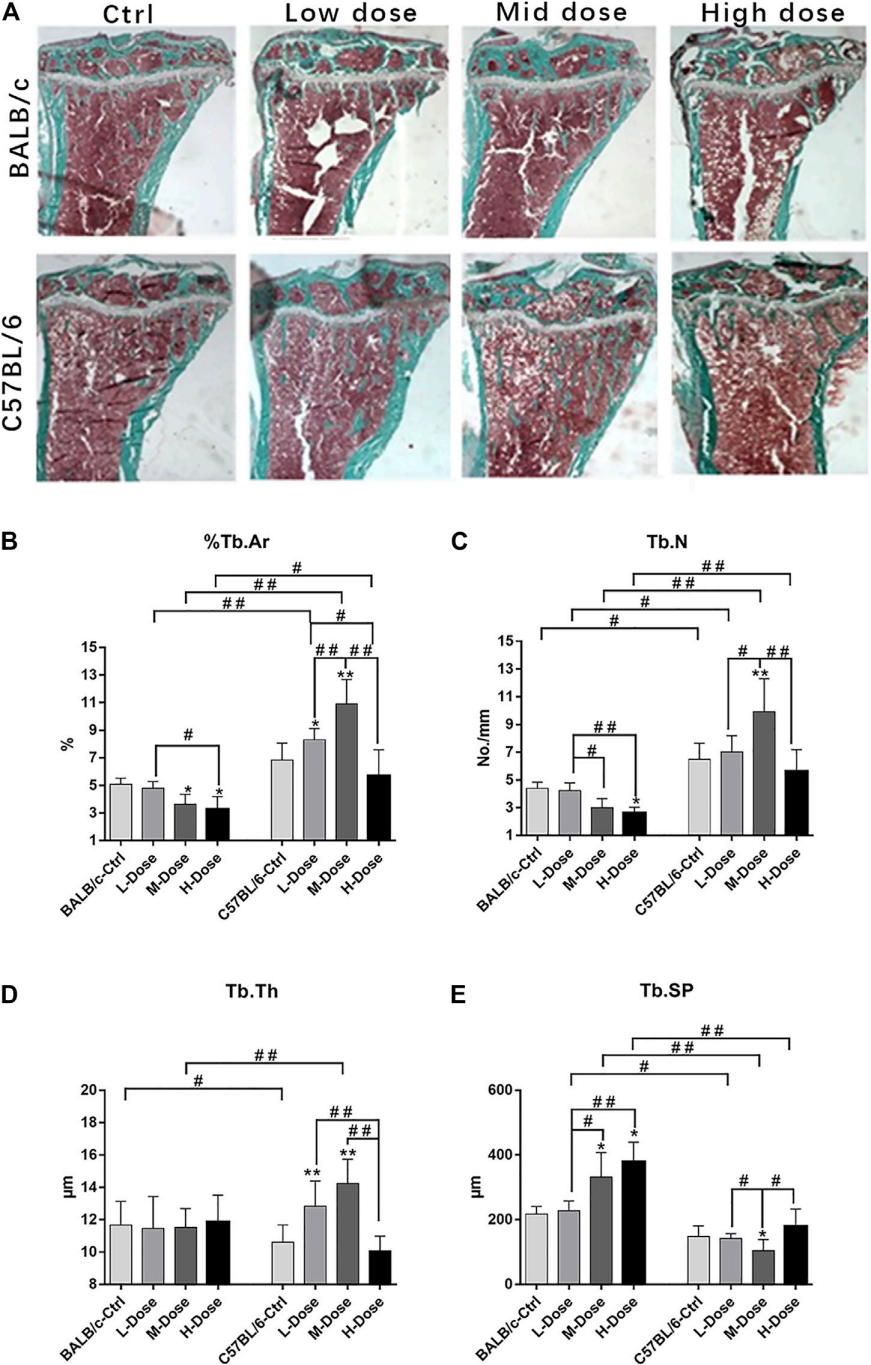
Changes of Trabecular bone of BALB/c and C57BL/6 in dexamethasone treatment. **(A)** Masson-Goldner Trichrome bone stains of the PTM showing the trabeculae bone loss causing by Dex. **(B–E)** Static parameters of PTM. *p < 0.05, **p < 0.01, significantly different from Ctrl; #*p* < 0.05, ##*p* < 0.01, significantly different between BALB/c and C57BL/6, n = 6. (%Tb.Ar, trabecular bone area percentage; Tb.N, trabecular number; Tb.Th, trabecular thickness; Tb. Sp, trabecular separation).

Indeed, BALB/c mice were more sensitive to the debilitating effects of Dex on trabeculae bone loss, despite a low-dose of Dex could only exert insignificant reduction in cancellous bone mass (Figure 1A). In contrast, C57BL/6 are largely tolerable to various dosages of Dex with minimal changes in bone mass except for a slight trend of dose-dependent loss (Figure 1B). Furthermore, we noted a reduction of cancellous bone in BALB/c mice PTM largely due to increased trabecular separation with decreased total trabeculae number (Figures 1C,E).

**FIGURE 2:**
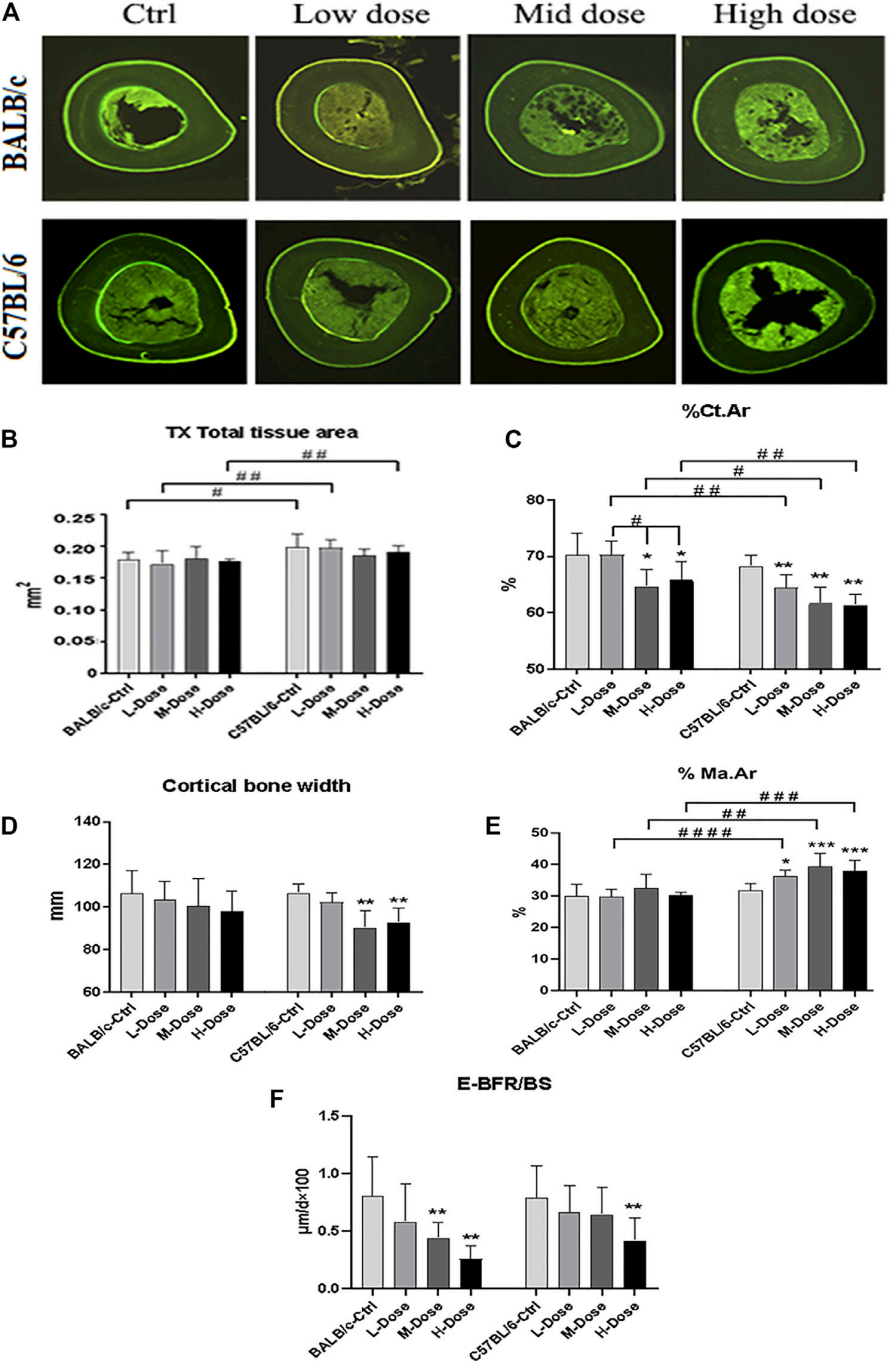
Changes of Cortical bone of BALB/c and C57BL/6 in dexamethasone treatment. **(A)** Images of TX transverse section fluorescence detection (injected with calcein). **(B–E)** Static parameters of TX. **(F)** Dynamic parameters of TX. *p < 0.05, **p < 0.01, significantly different from Ctrl; #*p* < 0.05, ##*p* < 0.01, significantly different between BALB/c and C57BL/6. (%Ct.Ar, percentage cortical area; %Ma.Ar, percentage marrow area; E-BFR/BS, cortical bone endosteal formation rate).

In addition, there is almost no differential impact in Dex treatment towards the cortical bone components (Figure 2A). The total tissue area remained the same as revealed from TX (Figure 2B).% Ct. Ar and cortical bone width were decreased (*p* < 0.05) while % Ma. Ar was increased (*p* < 0.05) in C57BL/6 mice compared to the control group (Figures 2C,E). Dynamic parameter of cortical bone also demonstrated a decrease in bone formation in endosteum in both strains (*p* < 0.01) (Figure 2F). Finally, we also confirmed that Dex exerted no effect in chondrocyte differentiation in the growth plates, bearing similar histomorphological features between these two strains. (Supplemental Figure S1). Therefore, our *in vivo* data confirmed BALB/c and C57BL/6 mice had recapitulated the iatrogenic effect of GC despite with a differential sensitivity towards the prolonged drug treatment likely due to their diverse genetic background.

### Multiple Comparison of Transcriptomic Profiles Identifies Dex-Induced Co-expression Network in Osteoblasts

To explore the transcriptomic differences contributed to various response to Dex caused by genetic background, we compared the expression profile of calvaria cultures among the previously reported CC mice, also importantly COL3.1-expression gated osteogenic progenitors from C57BL/6 across time; together with our two common laboratory strains of inbred mice (BALB/c and C57BL/6) with or without Dex administration after day 10 of mineralizing treatment *ex vivo*. Of note, CC mice would serve as ideal comparator to our experimental groups since one of their initial founder parent strains was the C57BL/6 line.

**FIGURE 3:**
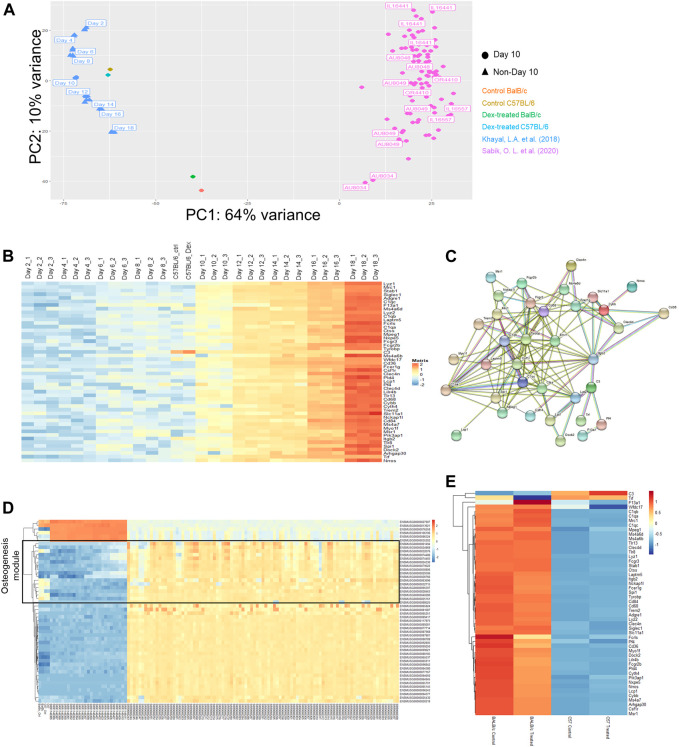
Multiple comparison of transcriptomic profiles. **(A)** Principal component analysis (PCA) plot for expression profiles of Day 10 *ex vivo* mineralizing osteoblasts from a collection of CC mice lines adapted from Khayal et al. (blue), C57/BL6 (brown/cyan) and BALB/c (red/green) mice with or without prolonged administration of Dex and differentiating Col3.1 + osteogenic stromal progenitors along osteogenic differentiation (Day 2 to Day 18) adapted from Sabik et al. (pink). **(B)** Expression changes in the top 50 genes contributing to the variance along PC2 listed out from top to bottom from the PCA plot. NB. Day 10 mineralizing C57/BL6 osteoblasts shared similar expression profile with mineralizing stromal progenitors upon Day 10 of their differentiation. **(C)** Protein-protein interaction network of co-expressed genes along PC2. **(D)** Expression changes in the top 50 genes contributing to the variance along PC1. NB. The top 50 genes clustered into the WGCNA module from Sabik et al. found with characteristic eigengene defined by osteogenic potential (denoted in black). **(E)** Expression profile of all genes in the protein-protein interaction network along PC2 in both BALB/c and C57/BL6 mice with and without prolonged administration of Dex.

With the advantage of sharing the tissue of origin, we performed principal component analysis (PCA) in the expression profiles via a dimension reduction approach to explain underlying variation amongst data-points. We observed the spread of expression profile of all CC mice with various genetic markup across the PC2 panel, despite only accounting intrinsically for 10% of total variance (Figure 3A). When locating all data-points as of day 10 for mineralization treatment (only data points indicated in circles), we noticed that all RNA-seq profile for mice model not being descendant of those CC breeds fall outside the spread but succumb to scatter across the spread in the PC1 panel, which accounted for 64% of the variation (Figure 3A).

From that PCA analysis, the spread of blue data-points from Khayal et al. were also included to account for variations along stromal cell differentiation from C57BL6 mice culture enriched exclusively with COL3.1 + osteogenic stromal progenitors. Intriguingly, a coherent top-to-bottom spread in expression profiles along PC2 was found like that reported in CC mice (Figure 3A). Notably, the spread in CC mice expression profile along PC2 is correspondingly reflective of inherent efficacy of stromal cell differentiation potential as it was previously reported from Sabik et al. that there is a tendency of increasing mineralization in these CC mice from top to down along PC2 (Figure 3B).

It will therefore be interesting to firstly look for gene ontologies of candidate genes accounting for the differential expression along the variance of PC2. It was found that a list of 50 genes were either co-expressed or silenced together in individual datasets (Figure 3B). Surprisingly, an overwhelmingly 35 of them were previously defined to fall within the module with eigengene defining immune response. There is a clear trend of increase in these immune-related genes in the stromal progenitors from day 2 towards day 18, where the protein-protein interactions among these genes marked up a co-expressed, interactive network with predictive value of the tendency of bone mineralization among CC mice calvaria osteoblasts (Figure 3C). Such inverse expression-mineral content relationship invites our further study in how such co-expression network drove the corresponding stromal cell differentiation.

In contrast to that of PC2, the spread along PC1 explained more variation among data-points (Supplementary Figure S2). Noticeably from the PCA, our C57BL/6 calvaria culture expression profile faithfully resembles that profile of Day 10 mineralizing stromal progenitors (Figure 3A), indicative of the predictive nature of PC1 in aligning expression profile from the same genetic background. Likewise, it can also be confirmed that the expression profile of BALB/c mice lie approximately midway in the genetic variation between the two datasets of mouse calvaria expression profiles. In parallel, there is an agreeably smaller spread in intrinsic background differences among those CC mice along PC1 where their data-points clustered together as expected. It should be reminded that despite C57/BL6 served as one of the 8 founder breeds for descendant CC pure breeds, none of these mice descendants carry an expression profile that blend in with data-points obtained from that C57/BL6 background.

We were therefore eager to study the underlying co-expressed gene network governing this PC1 spread (Figure 3D). One must also observe that the top-enriched 20 genes unmistakably fall within the module with eigengene defining osteogenesis. To sum up, PC1 explains a large baseline difference in intrinsic osteogenic potential of calvaria cultures while PC2 accounts for the subtle but significant co-expression changes along a panel of genes implicated in affecting efficacy in stromal cell differentiation, which invite further studies in the action of its underlying gene network.

Furthermore, we were also concerned with whether such variant baseline osteogenic potential shall give rise to differential sensitivities for GC towards osteogenesis. Since our drug treatment versus control remained at the closest proximity to each other, it was clearly indicated that intrinsic genetic differences defining the PC1 spread already lead a larger effect size that masks over the incremental horizontal vector gap under prolonged Dex administration. Hence, most of the discrepancies in expression profiles of isolated calvaria could again only be explained on the grounds of genetic background. Yet, it could nonetheless be confirmed that there was a significant larger vertical vector gap between data points along the PC2 in BALB/c compared to that in C57/BL6.

As mentioned above, it was confirmed that most of these PC2 genes belong to the same module defined by an immune-related eigengene based on weighted gene co-expression network analysis (WGCNA). These gene had also been subjected to gene-gene interaction network clustering, from which we had successfully constructed the stromal cell differentiation network (Figure 3E). Revisiting the gene expression of the entire network collectively, we can extrapolate a more significant expression changes in the BALB/c mice between the calvaria culture with or without treatment with Dex compared to that between the cultures isolated from C57BL/6 (Figure 3E). Of note, the top DEGs within this network are the murine model specific Fcrls and Cd36, both of which belongs to the scavenger receptor B family.

### Prolonged Dex Treatment Prevented Osteogenic Differentiation and Proliferation in Mice

**FIGURE 4:**
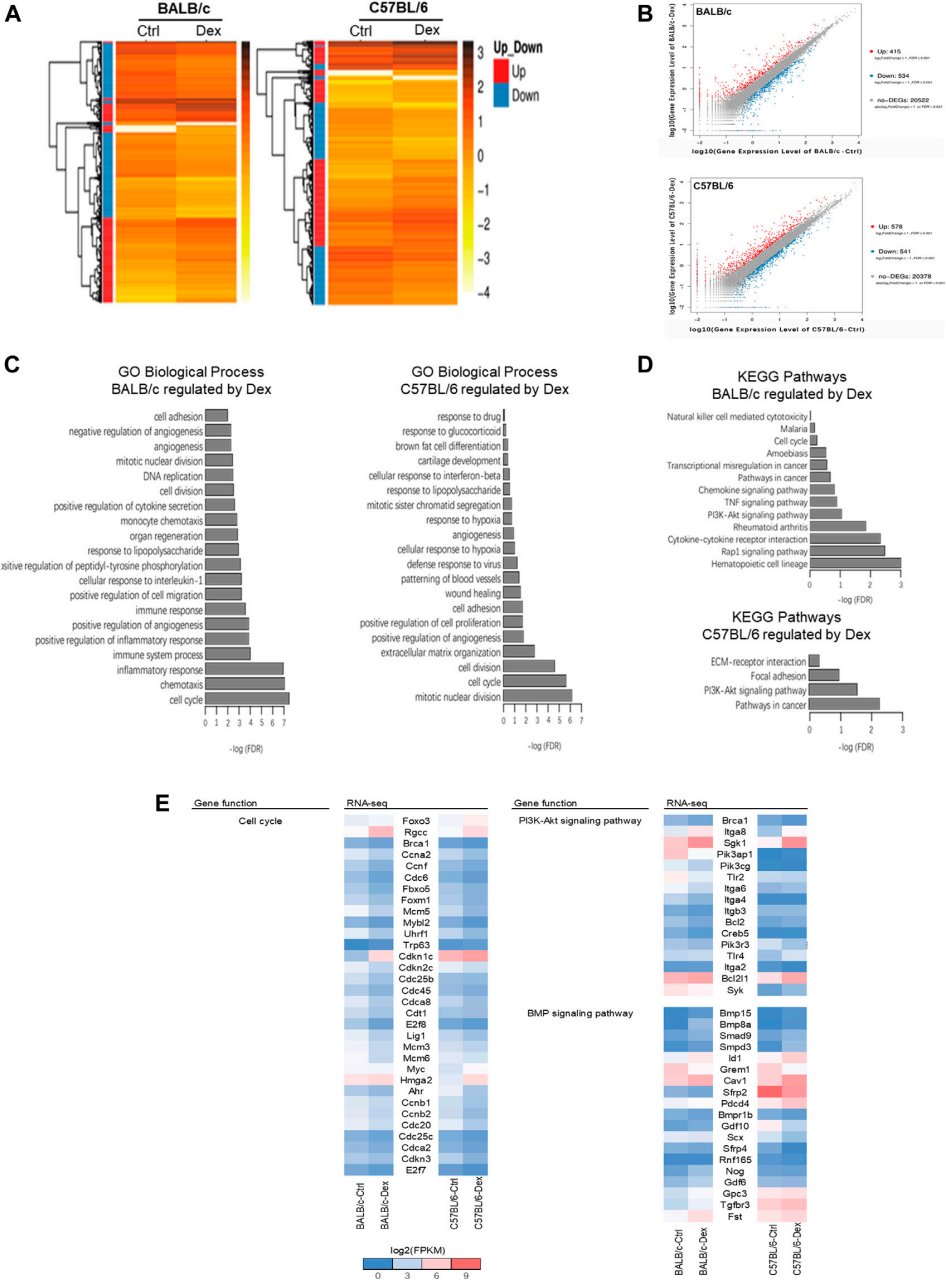
Transcriptomic analysis of genes regulated by dexamethasone in mouse osteoblasts of two strains. **(A)** Heatmap of DEGs in Dex-treated mouse osteoblasts. **(B)** Scatter plot of log10 FPKM values of genes expressed in Dex-treated osteoblasts versus untreated osteoblasts. The red dots and blue dots identify that are differentially expressed at log2FoldChange ≥ with FDR ≤0.001 between untreated and Dex-treated MSCs (red dots represent the up-regulated genes, blue dots represent the down-regulated genes, gray dots represent the non-DEGs). **(C)** Top 20 GO Biological process terms of Gene ontology analysis of Dex-regulated genes. **(D)** KEGG pathway analysis enriched signaling pathways altered by Dex. **(E)** Gene expression shown in log2(FPKM+1) of genes relevant to PI3K-Akt and BMP signaling pathways for untreated and Dex-treated osteoblasts of BALB/c and C57BL/6.

Our pair-wise comparison with or without drug treatment in both inbred mice strains pinpointed a collection of DEGs as a result in Dex pre-conditioning (Figure 4A). Compared to untreated BALB/c osteoblast, 949 DEGs (415 up-regulated and 534 down-regulated) were detected after Dex treatment; while for the C57BL/6 osteoblasts, there were 1119 DEGs (578 up-regulated and 541 down-regulated) were detected (Figure 4B). GO analysis demonstrated cell cycle, angiogenesis, cell adhesion, cell division, mitotic nuclear division, positive regulation of angiogenesis, positive regulation of cell migration and response to lipopolysaccharide commonly observed to be associated with Dex preconditioning in both BALB/c and C57BL/6 primary osteoblasts (Figures 4C,D). Most of these genes fall under PI3K-Akt signaling pathway in both BALB/c and C57BL/6 osteoblasts.

Nonetheless, the biological processes most commonly associated with the drug treatment differs between the two mice strain, with cell proliferation and extracellular matrix organization specifically regulated in C57BL/6 osteoblasts, while chemotaxis, immune system process and inflammatory response were top-hits in BALB/c osteoblasts instead (Figures 4C,D). In line with these processes, DEGs were clustered within focal adhesion and ECM-receptor interaction pathways specifically regulated by GC in C57BL/6 osteoblasts, while the TNF signaling pathway and other chemokine/cytokine signaling pathway, notably those belonging to the bone morphogenetic protein (BMP) superfamily, were specifically regulated by GC in BALB/c osteoblasts.

When taken together, we believe Dex regulates osteoblasts cell cycle, cell division and PI3K-Akt signaling pathway in both BALB/c and C57BL/6, while BMP signaling most enriched under the action of Dex in BALB/c osteoblasts which might partially explaining GC-induced bone loss in BALB/c mice (Figure 4E, Supplementary Table S1, S2). As such, we cross-validated known DEGs of BALB/c and C57BL/6 belonging to BMP signaling pathway implicated in osteogenesis for further investigation (Figure 4E and Supplementary Table S3). Several genes promote bone formation such as Smpd3 and Pdcd4 were increased in a higher fold-change by GC in C57BL/6 osteoblasts than in BALB/c osteoblasts, while bone formation inhibitors Scx and Sfrp4 were decreased in a higher fold-change by GC in C57BL/6 osteoblasts than in BALB/c osteoblasts. Compared with C57BL/6 primary osteoblasts, GC significantly upregulated the expressions of Nog, Fst, Gdf6, Gdf10 and Gpc3, which are well-known as osteogenesis inhibitors in BALB/c primary osteoblasts.

### Dex-pre-conditioned Mesenchymal Stem Cells Manifested Features Recapitulating Glucocorticoid Induced Osteoporosis

Our prolonged Dex treatment in animal model effectively recapitulated symptoms and expression profile changes associated with GIO. Nonetheless, natural variance across various mouse strains investigated during mineralizing treatment demonstrated substantial natural variation that masks over the subtle pathway changes along Dex treatment. Since collective increment of individual effect sizes underlying genetic predisposition between these mice were not sufficient to mark up a unified locus associated with GC susceptibility, we turned to an alternative approach in gathering epigenetic and pharmacological data to provide biological inference for GWAS.

**FIGURE 5:**
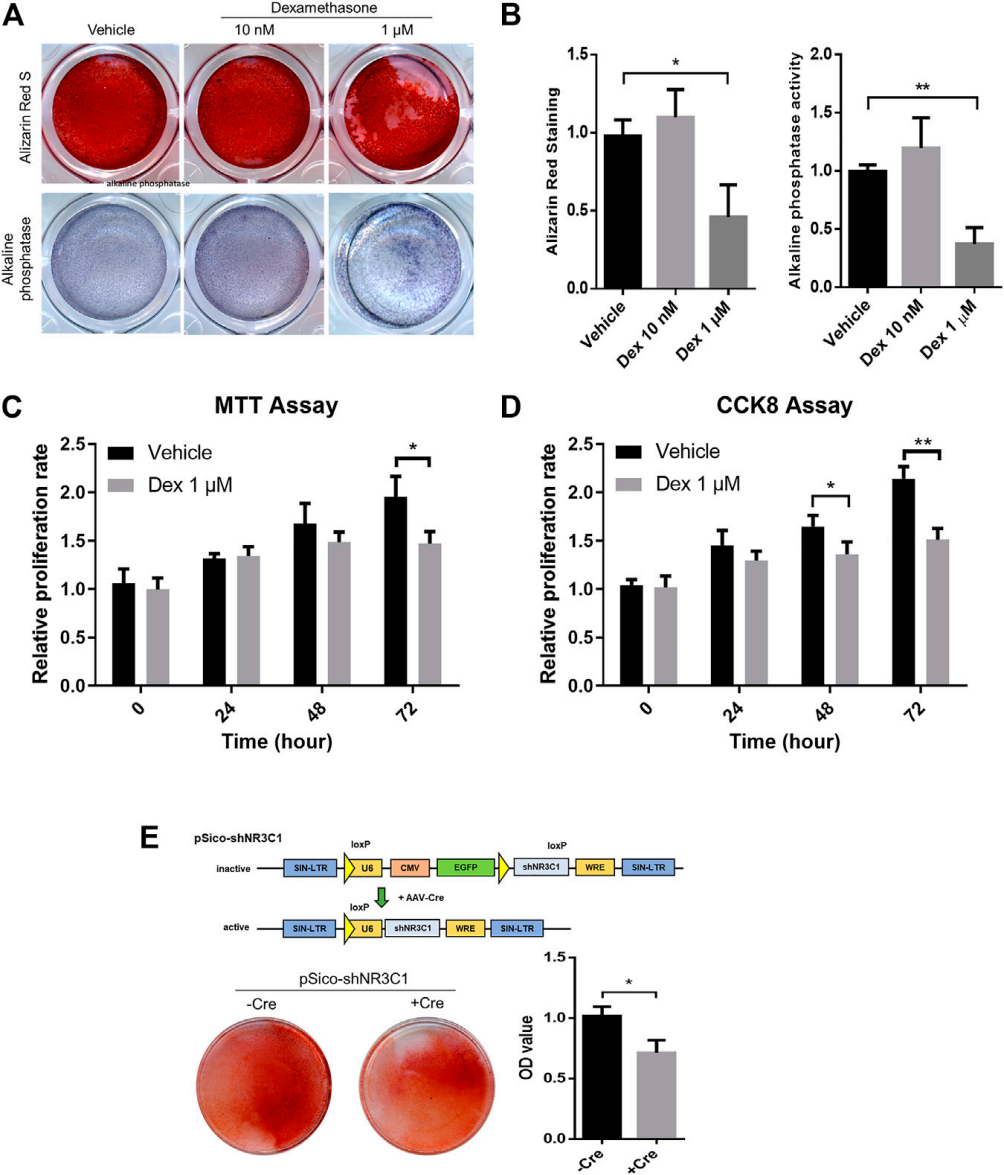
Micromolar levels of dexamethasone prevented osteogenesis and proliferation in pre-conditioned MSCs. **(A,B)** Alizarin Red staining and alkaline phosphatase staining of MSC-derived osteoblast culture. **(C,D)** MTT assay and CCK-8 assay of MSCs during the 72 h-interval of Dex preconditioning. **(E)** Cre-mediated silencing of NR3C1 rescued the osteogenic effect of MSCs which had subjected to Dex pre-conditioning. *p < 0.05, **p < 0.01, significant between groups.

To begin with, MSCs with osteogenic potential were allowed to pre-condition in either vehicle control, 10 nM or 1 μM Dex for 72 h before subjected to osteogenic differentiation (Figure 5A). There is a slight enhancement in osteogenic differentiation for MSC-derived osteoblast culture under a 20-days protocol when subject to Alizarin Red staining. However, micromolar levels of Dex did not promote strong osteogenic differentiation as expected. We had repeated the same set of experiment probing into the alkaline phosphatase (ALP) activity and had come up with the same conclusion (Figure 5B).

Micromolar levels of Dex is way beyond the fluctuating ranges of the normal hormonal level as in GC. Therefore, we speculated an overwhelmed GC signaling when MSCs were pre-conditioned with such high levels of GCs. By cross-referencing both MTT assay and CCK-8 assay, we observed significant reduction in proliferating MSCs during the 72 h-interval of Dex preconditioning (Figures 5C,D). Taken altogether, GC seemed to have imposed an overkill effect in osteogenic activity after pre-conditioning.

We were satisfied with our GC-disrupted osteogenic human MSCs model at such desensitizing Dex level, i.e., planned as the experimental set-up intended to simulate the iatrogenic effect of GC-induced osteoporosis. To confirm that GC prevented osteogenesis through the actions of its nuclear receptor NR3C1, therefore we performed lentiviral induction of pSico-shNR3C1 in MSCs followed by preconditioning in the same concentration (Figure 5E). The plasmid was adopted for the specific reversion of the hairpin-mediated knockdown effect of the nuclear receptor with transient Cre expression. Results clearly demonstrated that Cre-mediated silencing of NR3C1 rescued the osteogenic effect of MSCs even when subjected to Dex pre-conditioning (Figure 5E). Since we had demonstrated the involvement of NR3C1 in previous experiment, we had ruled out non-specific pharmacological inhibition of receptor proteins instigated by Dex.

### Epigenetic Marker Change in Mesenchymal Stem Cell-Derived Osteoblasts Identified *cis*-Expression Quantitative Traits Loci of Glucocorticoid Induced Osteoporosis

**FIGURE 6:**
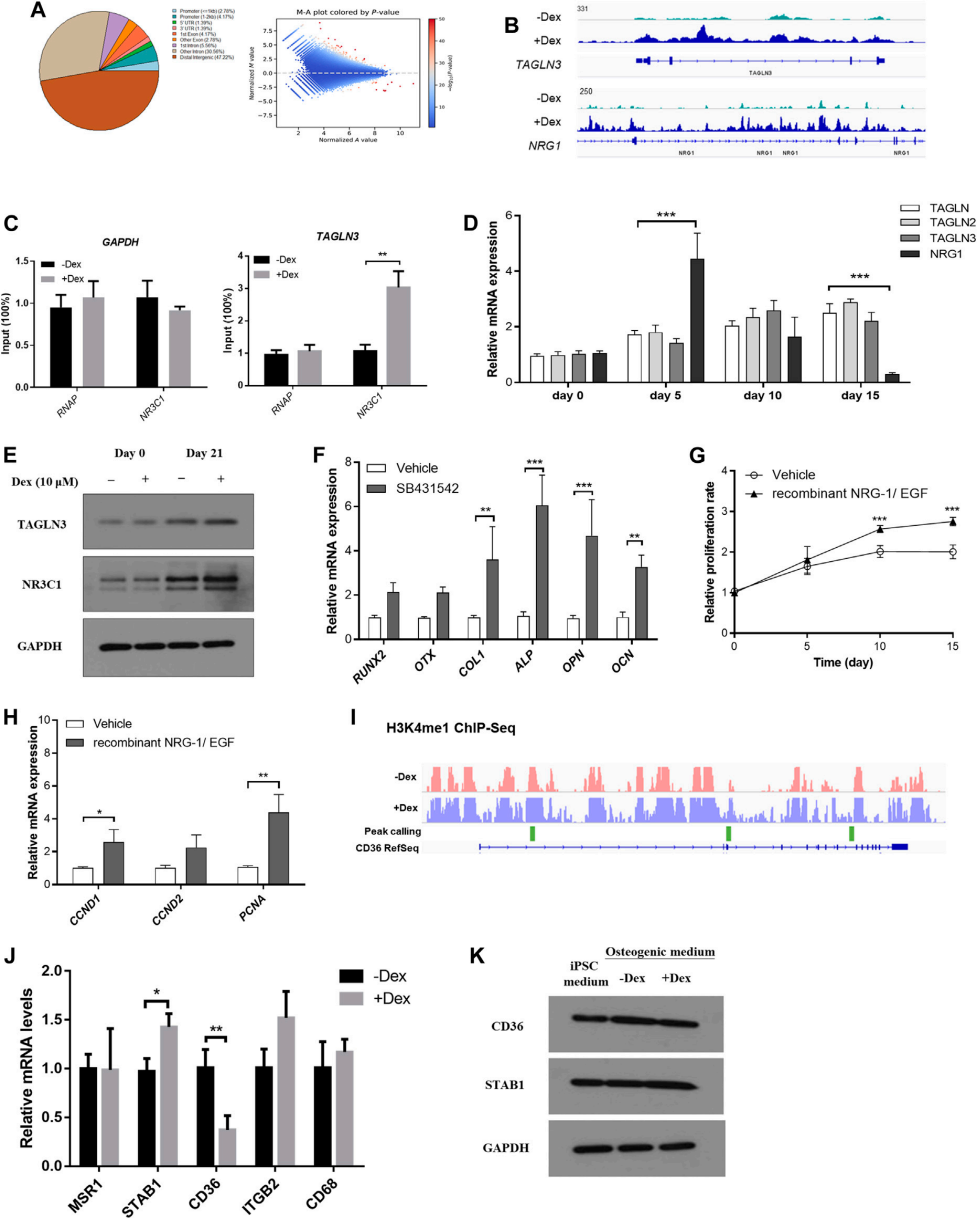
Chromatin remodelling led to heightened enhancer accessibility to GC signaling in pre-conditioned MSCs. **(A)** Distribution of identified peaks MSC-derived osteoblasts with or without Dex treatment. **(B)** TAGLN3 and NRG1 showed differential peaks between vehicle-treated and those pre-conditioned with Dex. **(C)** CHIP-PCR of TAGLN3 and NR3C1 in MSC-derived osteoblasts. **(D)** The gene expression of TAGLN members and NRG1 along osteogenic differentiation. **(E)** The protein expression of TAGLN3 and NR3C1 along osteogenic differentiation. **(F)** Osteogenic transcription factors gene expression in pre-conditioned MSCs with or without co-treatment with SB431542 (TGF-beta inhibitor). **(G,H)** The proliferation and gene expression of the co-culturing of pre-conditioned MSCs with either recombinant NRG-1/EGF (NRG-1 agonists). **(I)** Representative differential peaks of CD36 between vehicle-treated and those pre-conditioned with Dex. **(J,K)** mRNA and protein expression levels of CD36 and its associated genes. *p < 0.05, *p < 0.01, significantly different from Ctrl.

The human stromal cell osteogenic model had been adopted as a reliable model in studying osteogenesis. In line with the findings summarized from our mouse model, we hypothesize that GC pre-conditioned MSCs shall result in modifications within the epigenetic landscape which eventually give rise to variable extent of bone mineralization. In fact, there is a global induction of enhancer accessibility by performing chromatin-immunoprecipitation of the H3K4me1 mark, a unified epigenetic mark reflecting the enhancer regions (Figure 6A).

Furthermore, we therefore summarize the differential peak calling in the epigenetic mark H3K4me1 which may result in programmable tissue-specific chromatin accessibility which indirectly affect transcription factor binding in enhancer sites (Figure 6A). Enlightened by our previous mice experiments, we understood that cell proliferation through PI3K-Akt pathway and the BMP superfamily signaling pathways are involved in GC-induced osteogenic changes. We therefore tested whether these called peaks were functionally relevant to our GC-induced changes.

By focusing on those belonging to the transgelin (TAGLN) family, most notably member 3 (TAGLN3) (BMP superfamily signaling pathway) and neuregulin 1 (NRG1) (PI3K-Akt pathway) as they were also previously reported to play a role in MSC-derived osteogenesis (Figure 6B). We confirmed significant relaxation in selected regions of chromatin spanning their corresponding promoter with enhanced RNA polymerase and NR3C1 occupancy normalized to housekeeping gene GAPDH as confirmed by ChIP-PCR (Figure 6C). We monitored the gene expression of TAGLN members and NRG1 along osteogenic differentiation. Interestingly, TAGLN members were induced along the time-course of osteogenesis while there was a spike in NRG1 expression after Dex pre-condition followed by a gradual reduction along osteogenesis. In contrast, mild expression changes in these genes were observed in vehicle-treated only MSCs without Dex pre-conditioning (Figure 6D). Besides, we had also carefully validated the changes in protein expression of NR3C1 and TAGLN3 under the influence of DEX pre-condition (Figure 6E).

Osteogenic transcription factors, including RUNX2, OTX and osteoblast marker COL1, ALP, OPN and OCN were examined in pre-conditioned MSCs with or without co-treatment with SB431542 (TGF-beta inhibitor). Surprisingly, co-treatment with TGF-beta inhibitor (TAGLN pathway antagonist) in pre-conditioned MSCs enhanced osteogenesis marker expression (Figure 6F). Co-culturing of pre-conditioned MSCs with either recombinant NRG-1/EGF (NRG-1 agonists) also effectively promoted proliferation of the pre-conditioned MSCs resulting in partial rescue of osteogenesis (Figures 6G,H).

Besides, by leveraging inputs from epigenetic information, potential disease-relevant genes could be inferred from suggestive significant loci to provide the interpretation of pathological mechanisms. These differential epigenetic information before and after drug treatment shall render corresponding changes in *cis*-regulatory DNA histone marker changes as an alternative cost-effective approach to infer and predict trait-associated loci, in particular expression quantitative traits loci (eQTLs) directly or indirectly from GWAS results where sampling size remains inadequate.

In line with the findings in the mouse model, several of these genes were interconnected in the co-expressed network to the scavenger receptor Cd36, which itself belongs to the reported osteogenic core module. We investigated the differential H3K4me1 peaks along the Cd36 locus (marked in green). There is generally a more active open chromatin at the site in untreated osteoblasts compared to those pre-treated with Dex. Moreover, we confirmed a differential expression in Cd36 expression inferred from both mouse calvaria RNA-seq and qPCR analysis in the differentiated human MSCs (Figures 6I,J). To further reinstate the differential expression of scavenger receptors under the influence of Dex, we probed the changes in protein expression of CD36 and STAB1 in MSCs differentiated in osteogenic medium (Figure 6K).

Summarizing from above, we believe analyzing the global changes in H3K4me1 mark along osteogenic differentiation with or without Dex conditioning may suggest previously unknown targets such as TAGLN family in the TGF-beta signaling pathway and NRG1 in the EGF/mitogenic pathway potentially implicated in excessive GC-repressed osteogenesis and proliferation. Furthermore, we also identified a core-like scavenger receptor Cd36 interaction network responsible for stromal cell osteogenic differentiation which would effectively be modified under the actions of GC, potentially serving as a proxy biomarker for the idiosyncrasy in GIO.

## Discussion

Despite the wealth of genetic signals, the genes involved and the underlying mechanisms through which these associations affect osteoporosis remain largely unknown (Sabik and Farber, 2017). Past research cited BMD as the most clinically relevant heritable risk factor in assessment of osteoporosis, rendering dual-energy X-ray absorptiometry (DEXA) as an expensive but indispensable tool in bone disease diagnosis (Choi, 2016). Indeed, symptoms of bone loss with unknown etiology are prevalent in patients suffering from significant height loss or post-transplant, also in women with onset of menopause before age of 45 where DEXA is almost indispensable for disease assessment (Sözen et al., 2017).

Bone density screening with DEXA is also recommended for individuals with conditions that predispose to bone loss, which include hormonal type cancer treatments, suffering from parathyroid or thyroid disorders and particularly after prescription of steroid medications (Delitala et al., 2020). Femoral head osteonecrosis would also occur in individual patients given only a prolonged low-dose GC regime, or none such even at prolonged high dosage, casting a doubt in such pharmacological idiosyncrasy (Li et al., 2004). One may argue GC prevented both calcium absorption from the gastrointestinal tract and renal tubular calcium reabsorption therefore minimized mineral deposition. The debate is less persuasive given that calcium supplement alone is not adequate to restore bone density.

Similarly, high GC level was known to suppress gonadotropin secretion which in turn enhanced bone resorption. Histochemical analysis of adult bone however only demonstrated enriched GCR expression either in pre-osteoblast resembling mesenchymal stromal cells or the mature osteoblasts but null in osteoclasts (Li et al., 2004; Pierote et al., 2018). Unfortunately, except for arguably debates in receptor polymorphisms, there is no single locus found to associate with GC-induced idiosyncrasy. As in human subjects where TBS score could possibly be estimated directly from heel ultrasound, histomorphometric analyses seemed to have suggested a preferential loss of cancellous bone in the trabeculae for both common inbred mouse lines with variable sensitivities.

Our data do conform to such line-specific characteristic trabecular loss as the aftermath of a GC pre-conditioning period. Our current study highlighted that each strain exhibited a characteristic skeletal location, magnitude, and dose-dependent bone loss in response to Dex administration. Notably, there is a stark contrast in the severity of trabeculae loss between the animal lines. C57BL/6 is the most common laboratory mouse strain selected as the source of the mouse reference genome and the favorited genetic background for most commercial-available global knockout mice. From literature report, the baseline bone morphologies of another common line BALB/c is relatively similar to that in C57BL/6 (Wergedal et al., 2005) yet there remains unexplained subtle differences among the two lines in bone physiology. For example, in C57BL/6 mice, 6 weeks of loading resulted in lower stiffness of loaded tibiae versus contralateral controls, while in BALB/c mice, stiffness was not affected at all by loading (Holguin et al., 2013). Indeed, the structural, densitometric, and mechanical properties were different between aging BALB/c mice and C57BL/6 mice (Willinghamm et al., 2010).

Our current research initially sought to study the natural variance across common breeder strain to the collaborative cross using the same setting with or without prolonged high dose Dex treatment. To justify genetic background differences were responsible for these subtle idiosyncratic changes, we compared RNA-seq data obtained from *ex vivo* mineralizing osteoblast from animals with or without administration with high dosage of Dex. Collaborative Cross (CC) mice osteoblasts were included as control, which comes from a multiparent panel of recombinant inbred (RI) mouse strains built in attempt to enhances reproducibility and integration of data collected across time and conditions. The divergent allelic combinations and novel epistatic interactions of CC mice yield a spectrum of phenotypic variation with low levels of long-range disequilibrium for high resolution trait locus mapping (Welsh et al., 2012).

These lines were specifically designed to overcome the limitations of existing mouse genetic resources in studying genotype-phenotype correlations caused by combinatorial allele effects in complex traits. The CC mice models capture approximately 90% of common genetic variants through possessing eight distinct haplotypes at any genomic locus (Roberts et al., 2007). From our data, these mice expression profile varies with each other to a slighter extent along PC1. In contrast, the other common inbred mouse strains demonstrate a much larger extend of variance in their genetic mark-up along the PC1, compared to those transcriptional landscape even after given drug treatment. The involvement of a collection of osteogenic core module genes in defining PC1 confirmed the differential baseline characteristics that masked over Dex-induced changes.

Our *in vivo* assay demonstrated significant GC-induced trabecular bone loss had been found in BALB/c strain in both middle-dose and high-dose groups. However, no significant bone loss was shown in C57BL/6 mice trabecular bone, in contrast, significantly trabecular bone mass increase was shown in low and middle-dose groups. Regarding the cortical bone, the influence of GC in two strains was consistent, which was to inhibit the bone formation in both endosteum and periosteum, resulting in the bone total area unchanged, but the % Ct. Ar decreases. This suggests that BALB/c mice were more sensitive to GC and were governed by subtle differences in genetic background which limited their responsiveness towards the drug, despite bearing only one unified pharmacological target.

Besides, our PCA analysis included relevant data-points extrapolated from a collection of CC mice mineralizing osteoblasts and that of osteogenic differentiation of C57/BL7 stromal precursor. In fact, we observed a strong resemblance in our C57BL/6 mineralizing osteoblast to that of stromal precursor amidst day 10 osteogenic differentiation but varies tremendously to those of the CC mice. To our dismay, there is only little post-treatment variation in gene expression for those C57/BL6 mice administered with high dosage of Dex. Similarly, BALB/c do not fall within a similar expression profile to mineralizing osteoblast from CC mice nor to those form C57/BL6. Of note, C57BL/6 but not BALB/c were among one of the 8 founder lines to all CC lines.

Indeed, the realms for the natural variance across different inbred mouse lines were already much higher than that compared to with drug treatment as demonstrated from the supposed BALB/c outgroup. Nonetheless, the divergence in expression profile from BALB/c with and without Dex administration remains more pronounced than that between data-points of C57/BL6 mice, further elaborated the more severe phenotype as seen in this inbred line. We therefore turned to the human MSCs osteogenic model to investigate the genetic loci associated with severity of Dex-induced pharmacological effects imposed to osteoblasts as a useful predictor to heritable fracture risk in GIO.

The largest GWAS compiled from DEXA-derived BMD measures only include around 33,000 individuals (Amblard et al., 2003), which severely compromised detection of risk loci, especially in complex traits where effect sizes for most loci were minimal. The ultrasound-derived eBMD values were also found to be highly heritable (Turner et al., 2000; Judex et al., 2004; Bouxsein et al., 2005; Li et al., 2005), therefore its substitute for DEXA would have immensely populated the sample size to increase the power of the experiment. Since it was known that *cis*-eQTLs usually have much larger effect sizes than *trans*-eQTLs, we can expect that many of the biggest signals in GWASs are *cis*-regulators of core genes. Finally, the eBMD was also notably predictive to hip and spine fractures (Dovio et al., 2004), therefore we shall overlay the gene loci identified from these studies facilitated our hunt in *cis*-eQTLs contributing to GIO. Since one expects core genes to be co-expressed together in the same disease-relevant cell type, the logical approach to identify protein interaction relationship among genes after integrating the results of GWAS with co-expression networks to reflect the transcriptional programs associated with the trait of interest. The assorted haplotype in CC mice lines defined a spectrum of gene expression profile formed the basis for constructing a WGCNA network for osteoblasts (Sabik et al., 2020). From there, a clear co-expression eigengene module was enriched for genes with well-known roles in osteogenic activity after integrating eBMD GWAS information.

As in any other GWAS study, significant non-Mendelian variants are highly enriched in active chromatin such as promoters and enhancers as *cis*-regulatory gene loci correlated well with stromal cell osteogenic differentiation phenotypes. The alteration in enhancer histone marker H3K4me1 would therefore represent an additional layer of independent information to overlay the search of GIO-relevant gene loci, especially when differential histone mark was present in these osteogenic core genes when subjected to Dex pre-conditioning. We subsequently co-clustered the differential epigenetic signature in human MSCs with the RNA expression variations revealed from mice RNA-seq profile. These pathways were clustered as the key differentially expressed pathway altered between *ex vivo* osteoblasts with or without high dosage Dex-administered. Changes in gene transcription levels on the BMP pathway were therefore enriched in the cytokine-cytokine receptor interaction by the KEGG pathway analysis. Among DEGs in BMP signaling pathway, Nog, Fst, Gdf6 and Gpc3 level was significantly elevated by GC in BALB/c primary osteoblasts. Noggin and Follistatin were reported as inhibitors of BMP signaling (Hayashi et al., 2009; Kattamuri et al., 2012). It has been reported that Dex increased mRNA expression of several BMP antagonists, including Follistatin. Gpc3 and Gdf6, also known as BMP-13, were reported as an inhibitor of bone formation (Shen et al., 2009; Dwivedi et al., 2013).

We further validated a differential chromatin accessibility landscape in the TAGLIN family members and NRG-1 loci under Dex pre-treatment, while the same genes also demonstrated differential expression in mice with or without Dex treatment. In fact, we confirmed a differential GCR NR3C1 binding near the transcription machinery of TAGLN3 upon GC preconditioning, suggesting chromatin accessibility for transcription factor binding on enhancer is a potential missing link for differential transcriptomic changes. Since Dex also effectively promoted a transient surge of NRG-1 expression as a growth factor which diminished gradually afterwards, cell proliferation was halt at later time points along osteogenesis and gradually diminished. The high dose Dex-mediated upregulation of transgelin members by TGFbeta signaling effectively activated differentiation pathway and diminished cell proliferation.

In summary, we found that differential activation of PI3K-Akt and BMP pathways between the mice strains may be involved in the relative ease of enhancer mark accessibility instigated by GC. A unified primary activation of the TGF-beta signaling pathway through the TAGLN family of genes together with NRG-1 transient activation by GC on their *cis*-regulatory enhancer marks had in fact staged the secondary differential osteogenic potential towards GC. In fact, the downstream regulated pathway of the PC2 gene network in the human osteogenic model remains both the PI3K-Akt and the BMP signaling pathway.

Our last endeavor was to demonstrate that epigenomic marker change after pharmacological treatment can be used as cost-effective approach to enhance the inferences for GWAS results, especially in situations when few genome-wide significant loci are available. In fact, it was already proven that epigenetic and pharmacological data can be used to provide biological inference for GWAS (Björkegren et al., 2015; Xue et al., 2018; Mullins et al., 2021). It was previously defined that a gene could only serve as a core gene if and only if the gene product exerts a direct effect instead of indirectly acting on other gene products within the cellular and organismal processes to achieve an expected phenotype (Boyle et al., 2017; Liu et al., 2019). This also suggests that peripheral gene would account for a substantial component of the heritability of a common trait. The current report is the first demonstration that mesenchymal progenitors pre-conditioned with high dose GC shall induce histone modification leading to a relatively hindered osteogenic effect.

As a proof-of-concept, we were interested in whether Dex indeed resulted in modulating the co-regulatory PC2 gene network, with a particular focus on the top enriched gene ontology for scavenger receptor signaling depended on these genes. We adhered closely to confirm the gradient expression changes along this gene network in mice. We further adopted enhancer accessibility as a proxy marker to deduce the contribution of Dex during the osteogenic differentiation process, where CD36 was defined as the core-like scavenger receptor responsible for the action. Functional non-coding elements predicted from GWAS may imply peripheral genes responsive to GC within this omnigenic trait of osteogenesis governed by the core gene CD36 potentially affected by high-dose Dex pre-condition. Indeed, it was found that several genes in the PC2 co-expression network were previously implicated in the BMD GWAS, which includes Igtb2, Cd68 and Ms4a6d. Given their incremental effect size, they may also be defined as peripheral genes for GIO contributing to the reduced trabeculae mass phenotype defined by the omnigenic core gene CD36. In fact, we had indeed characterized the differential H3K4me1 changes in turn affected CD36 expression along their osteogenic differentiation.

Scavenger receptors were implicated in bone turnover subject to the cell type they were expressed. It was described in macrophages that MSR1 would mediate PI3K/Akt signaling to promote osteogenic differentiation of MSCs in co-culture system (Zhao et al., 2020). Likewise, another scavenger receptor Stab1 could be found in both osteoblast and osteoclasts, while it was deduced to affect osteoclast maturation during bone resorption (Kim et al., 2019). Indeed, both scavenger receptors were found to contribute to defining the variance along PC2.

Furthermore, it was found that the high-density lipoprotein (HDL) type B scavenger receptor (Scarb1) knockout in bone MSCs affected their potential to undergo osteoblast differentiation (Tourkova et al., 2019). Importantly, another study using the same Scarb1 KO mouse model reported a compensatory level of adrenocorticotropic hormone (ACTH) levels for GC homeostasis which in turn affected trabeculae mass. Due to this compensatory hormonal control, these Scarb1 null mice are not prone to osteoporosis in general but instead demonstrated higher bone mass associated with enhanced bone formation (Martineau et al., 2014).

In the current study, CD36/SCARB3 belongs to the PC2-defined stromal cell differentiation co-expression network while itself was characterized as a core gene within the module implicated in bone mineralization (Sabik et al., 2020). Aside from acting as an import protein for fatty acids, the integral protein itself also acts as a scavenger receptor for native and oxidized low-density lipoprotein (oxLDL) (Jay et al., 2015). It was reported in the specific CD36 knockout mice the gene would not result in changes in femoral nor tibial length (Kevorkova et al., 2013). Nonetheless, microCT revealed a lower bone mass phenotype in either gender of these mice with high trabecular separation and reduced trabeculae number compared to that in wild type. Corresponding changes in those bone marrow stromal cells and osteoblasts depleted of CD36 resulted in reduced cell culture expansion with low gene expression of both osteoblastic transcription factor Runx2 and Osx.

In favor of our current experiment, these histomorphological and biochemical changes along depletion of CD36 closely resembles that of features as observed in the BALB/c animals under prolonged administration of Dex. Also, it was specifically observed that in the expression profile of BALB/c *ex vivo* osteoblasts that the expression of CD36 shall be downregulated potentially giving rise to manifested phenotypes of GIO. It is also with this idiosyncratic response towards Dex which give a specific significant susceptibility of BALB/c to that in C57BL/6 mice instead. Since CD36 is protagonist of a mitotic crosstalk resembling that of SCARB1 in which it accelerates cell growth via the Src-mediated PI3K/Akt axis (Tao et al., 2015; Luo et al., 2021). It is therefore also expected that the suppressing of this gene likely reduced osteoblastic proliferation via this pathway. Those two genes that transcend their effects amplified along the interaction with network of co-expressed core genes, here being CD36 specifically, to reflect their underlying transcriptional programs associated with the trait of interest relevant to idiosyncratic susceptibility towards BMD changes leading to bone loss because of GC treatment.

## Conclusion

Our results clearly demonstrated differential sensitivity towards epigenetic changes along a unified GC-regulated osteogenic pathway may likely explain variant genetic susceptibility towards GC-induced osteoporosis. Finally, genetic loci associated with eBMD are strongly enriched for the targets of clinically relevant osteoporosis therapies, the identification of new genetic loci and the biological pathways they implicate may help scientists identify drug targets for the prevention and treatment of fragility fracture.

## Data Availability

The data presented in the study are deposited in the NCBI repository, accession number: PRJNA770448 and PRJNA770459.
