# Supplementary material for: A Core Omnigenic Non-coding Trait Governing Dex-Induced Osteoporotic Effects Identified Without DEXA
Source: Front Pharmacol. 2021 Nov 24;12:750959. doi: 10.3389/fphar.2021.750959 (PMC8651565; doi:10.3389/fphar.2021.750959)
Supplement: Supplementary file 1 [file DataSheet1.pdf]

Title：A core omnigenic non-coding trait governing DEX-induced osteoporotic effects identified without DEXA

Li Lu [1], Yanzhen Cai [1], Xiaoling Luo [1], Zhangting Wang [2], Sin-Hang Fung [2], Huanhuan Jia [1,4], Chi-Lam Yu [2], Wai-Yee Chan [2], Kai-Kei Miu [2]*, Wende Xiao [3]*

[1] School of Life Science and Biopharmacy, Guangdong Key Laboratory of Pharmaceutical Bioactive Substances, Guangdong Pharmaceutical University, Guangzhou, China;

[2] School of Biomedical Sciences, Faculty of Medicine, The Chinese University of Hong Kong, Hong Kong SAR, China;

[3] Department of Orthopedics, Guangzhou First People's Hospital, School of Medicine, South China University of Technology, Guangzhou, Guangdong, China;

[4] Guangdong Key Laboratory of Laboratory Animals, Guangdong Laboratory Animals Monitoring Institute, Guangzhou, China

* **Correspondence:** Wende Xiao, Department of Orthopedics, Guangzhou First People's Hospital, School of Medicine, South China University of Technology, Guangzhou, Guangdong, China. Phone: 86-139 2890 8180, E-mail: eyxiaowende@scut.edu.cn

* **Correspondence:** Kai-Kei Miu, School of Biomedical Sciences, Faculty of Medicine, The Chinese University of Hong Kong, Hong Kong SAR, China. Phone: (852) 6533-3196, E-mail: kelvinmiu@cuhk.edu.hk

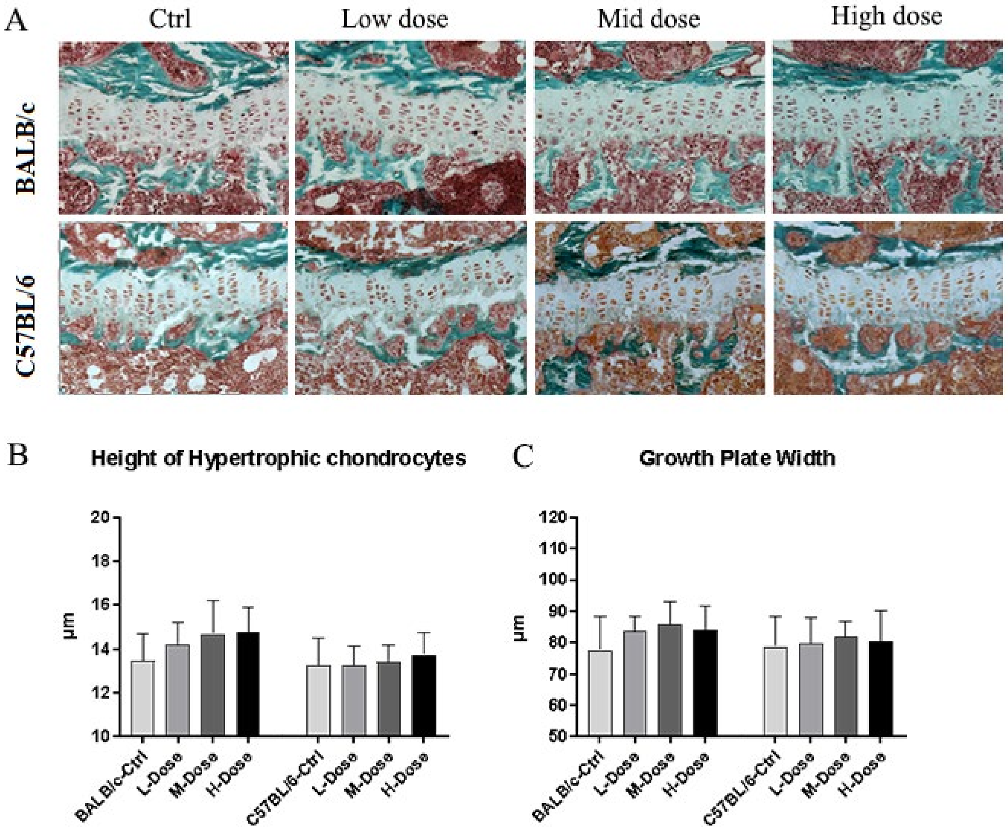

Figure S1. Changes of the Growth plate of BALB/c and C57BL/6 in dexamethasone treatment. (A) Masson-Goldner Trichrome bone stains of Growth plate. (B, C) Static parameters of Growth plate, **P*<0.05, ***P*<0.01, significantly different from Ctrl; #*P*<0.05, ##*P*<0.01, significantly different between BALB/c and C57BL/6.

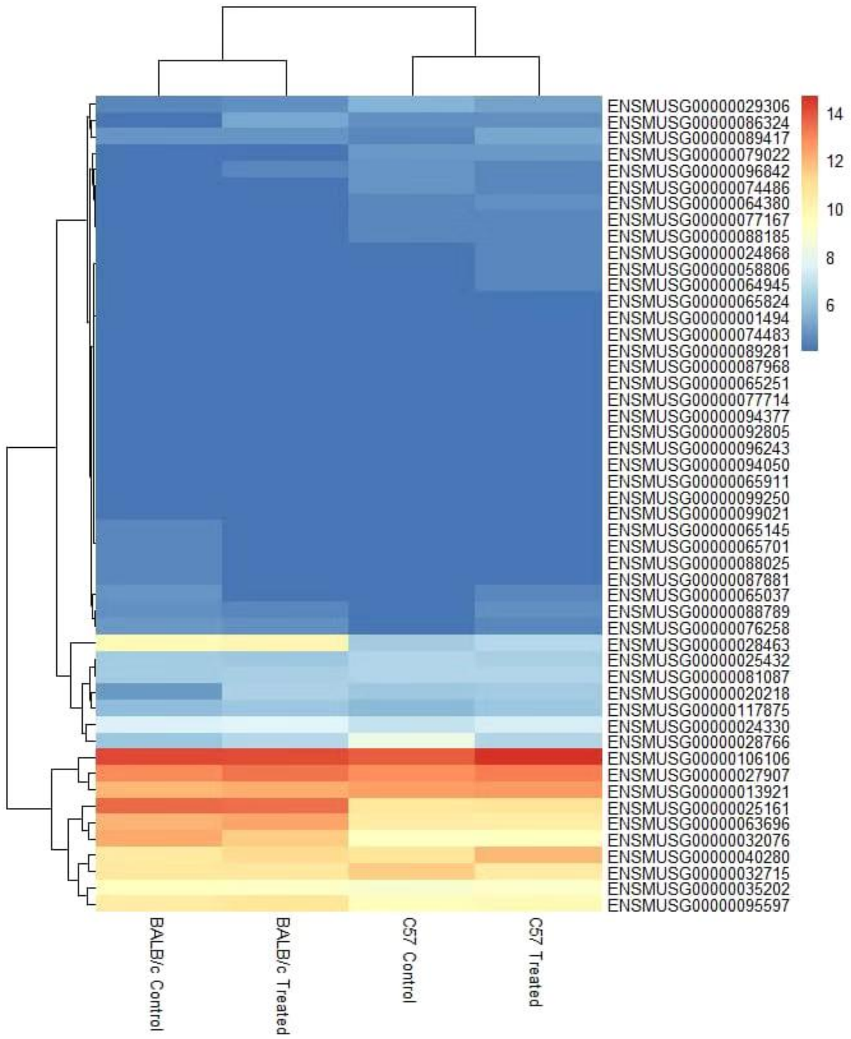

Figure S2. Expression of genes clustered in PC1. The expression of genes presented in PC1 were indicated in both BALB/c and C57BL/6 mice treated with DEX or not.

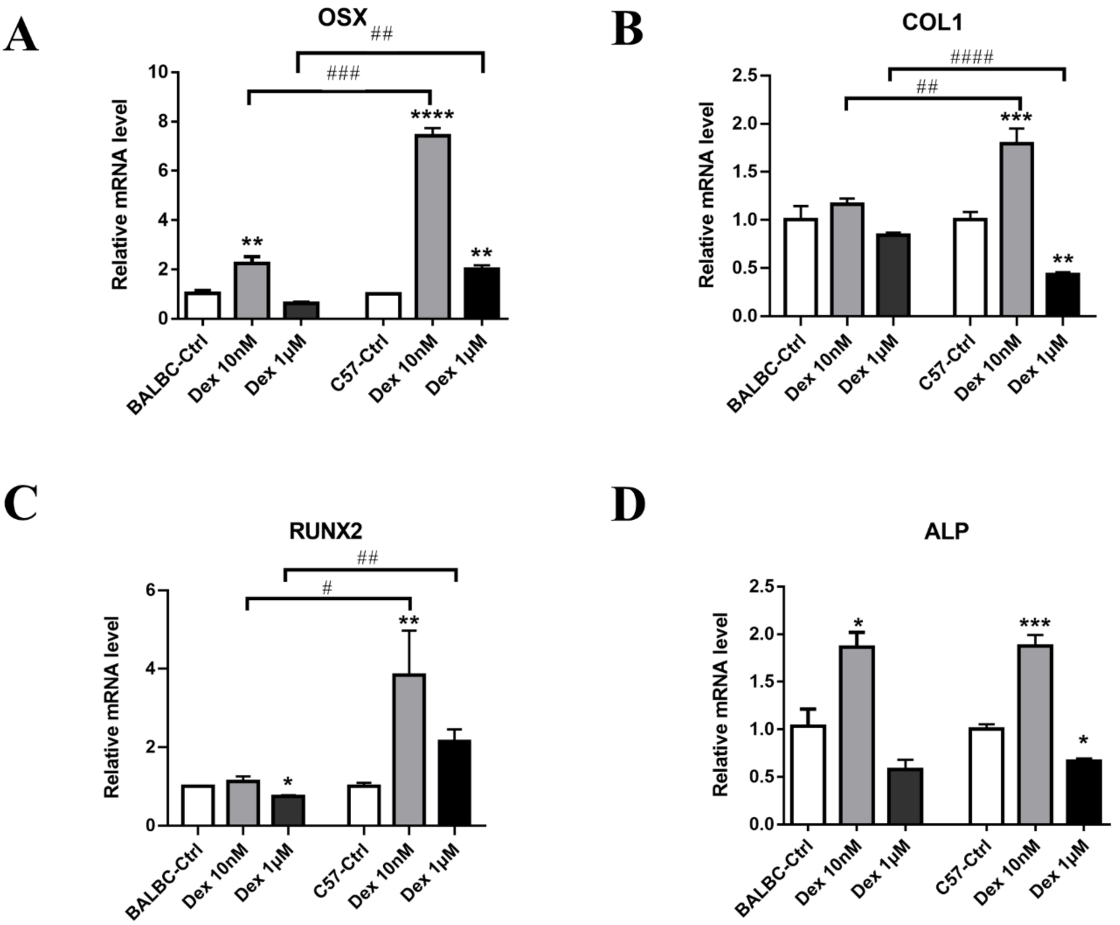

Figure S3. Osteogenic genes expression of mouse MSCs with or without pre-conditioned Dex treatment. (A-D) Relative mRNA expressions of osteogenic marker genes. For examining the osteogenic genes expression (OSX,COL1,RUNX2 and ALP) of mouse MSCs, DEX- or vehicle-treated mouse MSCs were subjected to the same 72h pre-conditioning followed by 72h treatment in the osteogenic medium. *P<0.05, **P<0.01, ***P<0.001, ****P<0.0001, significantly different from Ctrl; #P<0.05, ##P<0.01, ###P<0.001, ####P<0.0001, significantly different between BALB/c and C57BL/6.

Table S1. Effects of glucocorticoid excess on cell cycle

Gene symbol	Gene description	Gene Function	log2Fold Change (BALB/c _Dex)	log2Fold Change (C57BL/6 _Dex)
Ccna2	cyclin-A2	cell cycle stimulator	-1.13	-1.14
Foxo3*	forkhead box protein O3	cell cycle arrest	0.31	0.84
Cdkn1c	cyclin-dependent kinase inhibitor 1C	cell cycle arrest	3.81	0.67
Myc*	Myc proto-oncogene protein	cell cycle stimulator	-0.06	1.17
Cdc25b	M-phase inducer phosphatase 2	cell cycle stimulator	-1.26	-0.29
Cdt1	chromatin licensing and DNA replication factor 1	cell cycle stimulator	-1.14	-0.23
Cdkn3	cyclin-dependent kinase inhibitor 3	cell cycle arrest	-0.45	-1.52
Rgcc	regulator of cell cycle RGCC	cell cycle arrest	1.93	1.19
E2f8*	E2F transcription factor 8	cell cycle stimulator	-2.21	-0.99
Mcm3*	DNA replication licensing factor MCM3	cell cycle stimulator	-1.23	-0.53
Mcm6*	DNA replication licensing factor MCM6	cell cycle stimulator	-1.31	-0.45
Ahr*	aryl hydrocarbon receptor	cell cycle arrest	-0.76	-1.84

Brca1*	breast cancer type 1 susceptibility protein	cell cycle arrest	-1	-1.14
Ccnb1	cyclin B1	cell cycle stimulator	-0.71	-1.15
Ccnb2	G2/mitotic-specific cyclin-B2	cell cycle stimulator	-0.84	-1.42
Ccnf	cyclin-F	cell cycle stimulator	-1.41	-1.16
Cdc20	cell division cycle 20, cofactor of APC complex	cell cycle stimulator	-0.8	-1.55
Cdc25c	M-phase inducer phosphatase 3	cell cycle stimulator	-0.76	-1.14
Cdc45	cell division control protein 45	cell cycle stimulator	-1.11	-0.88
Cdc6	cell division control protein 6	cell cycle stimulator	-1.64	-1.23
Cdca2	cell division cycle-associated protein 2	cell cycle stimulator	-0.94	-1.01
Cdca8	cell division cycle associated 8	cell cycle stimulator	-1.39	-0.9
Cdkn2c*	cyclin-dependent kinase inhibitor 2C	cell cycle arrest	-1.11	-0.73
E2f7*	transcription factor E2F7	cell cycle stimulator	-0.8	-1.31
Fbxo5	F-box protein 5	cell cycle stimulator	-1.36	-1.14
Foxm1*	Forkhead box M1	cell cycle arrest	-1.06	-1.12
Lig1	DNA ligase 1	cell cycle stimulator	-1.4	-0.87
Mcm5*	DNA replication licensing factor MCM5	cell cycle stimulator	-1.65	-1.58
Mybl2*	myb-related protein B	cell cycle stimulator	-1.5	-1.64
Uhrf1*	E3 ubiquitin-protein ligase UHRF1	cell cycle stimulator	-1.59	-1.31

*Transcriptional factors. Values are the log2foldchange versus Control group. Gene Descriptionsources: KO

(KEGG ORTHOLOGY) Database and Nr (NCBI non-redundant protein sequences) Dataset.

Table S2. DEGs in PI3K-Akt signaling pathway regulated by dexamethasone

Gene symbol	Gene description	Gene function	log2Fold Change (BALB/c _Dex)	log2Fold Change (C57BL/6 _Dex)
Pik3ap1	phosphoinositide 3-kinase adapter protein 1	promote cell proliferation	-1.58	3.46
Itga4	integrin alpha 4	anti-apoptosis	-1.44	-0.10
Bcl2	apoptosis regulator Bcl-2	anti-apoptosis	-1.41	0.54
Creb5*	cyclic AMP-responsive element-binding protein 5	rescue cell death and promote proliferation	-1.45	-0.17
Itga8	integrin alpha 8	anti-apoptosis	1.40	2.39
Pik3cg	phosphatidylinositol-4,5-bisphosphate 3-kinase catalytic subunit gamma	anti-apoptosis	-1.03	-1.32
Sgk1	serum/glucocorticoid-regulated kinase 1	anti-apoptosis	1.45	2.64

*Transcriptional factors. Values are the log2foldchange versus Control group. Gene Descriptionsources: KO (KEGG ORTHOLOGY) Database and Nr (NCBI non-redundant protein sequences) Dataset.

Table S3. **DEGs in BMP pathway regulated by dexamethasone**

Gene symbol	Gene description	Gene function	log2Fold Change (BALB/c _Dex)	log2Fold Change (C57BL/6 _Dex)
Nog	noggin	inhibit bone formation	2.01	-0.15
Fst	follistatin	inhibit bone formation	1.19	0.37
Gdf6	growth differentiation factor 6	inhibit bone formation	1.07	-0.16
Gpc3	glypican 3	inhibit bone formation	1.17	0.25
Scx*	scleraxis	inhibit bone formation	-0.24	-1.66
Sfrp4	secreted frizzled-related protein 4	inhibit bone formation	-0.58	-4.71
Smpd3	sphingomyelin phosphodiesterase 3	promote bone formation	1.7	3.39
Pdcd4	programmed cell death protein 4	promote bone formation	0.37	1.07
Bmp15	bone morphogenetic protein 15	inhibit bone formation	4	3.7
Bmp8a	bone morphogenetic protein 8A	promote bone formation	6.2	5.17
Bmpr1b	bone morphogenetic protein receptor type-1B	promote bone formation	-0.81	-1.2
Gdf10	growth differentiation factor 10	inhibit bone formation	0.76	-2.18
Grem1	gremlin-1	inhibit bone formation	-1.36	-1.5
Id1	DNA-binding protein inhibitor ID1	promote bone formation	1.1	1.2
Sfrp2	secreted frizzled-related protein 2	inhibit bone formation	-0.73	-1.51

Smad9	mothers against decapentaplegic homolog 9	inhibit bone formation	1.2	1.28

*Transcriptional factors. Values are the log2foldchange versus Control group. Gene Descriptionsources: KO (KEGG ORTHOLOGY) Database and Nr (NCBI non-redundant protein sequences) Dataset.
